# Cell Electrical Impedance as a Novel Approach for Studies on Senescence Not Based on Biomarkers

**DOI:** 10.1155/2016/8484217

**Published:** 2016-10-12

**Authors:** Jung-Joon Cha, Yangkyu Park, Joho Yun, Hyeon Woo Kim, Chang-Ju Park, Giseok Kang, Minhyun Jung, Boryeong Pak, Suk-Won Jin, Jong-Hyun Lee

**Affiliations:** ^1^Department of Biomedical Science and Engineering, Gwangju Institute of Science and Technology (GIST), MEMS and Nano Systems Laboratory No. 317, Dasan Building, 123 Cheomdangwagi-ro, Buk-gu, Gwangju 61005, Republic of Korea; ^2^School of Mechanical Engineering, Gwangju Institute of Science and Technology (GIST), MEMS and Nano Systems Laboratory No. 317, Dasan Building, 123 Cheomdangwagi-ro, Buk-gu, Gwangju 61005, Republic of Korea; ^3^School of Life Sciences, Gwangju Institute of Science and Technology (GIST), Laboratory of Vascular Development No. 115, Life Science Building, 123 Cheomdangwagi-ro, Buk-gu, Gwangju 61005, Republic of Korea; ^4^Yale Cardiovascular Research Center, Department of Internal Medicine, Yale University, 300 George Street, New Haven, CT, USA

## Abstract

Senescence of cardiac myocytes is frequently associated with heart diseases. To analyze senescence in cardiac myocytes, a number of biomarkers have been isolated. However, due to the complex nature of senescence, multiple markers are required for a single assay to accurately depict complex physiological changes associated with senescence. In single cells, changes in both cytoplasm and cell membrane during senescence can affect the changes in electrical impedance. Based on this phenomenon, we developed MEDoS, a novel microelectrochemical impedance spectroscopy for diagnosis of senescence, which allows us to precisely measure quantitative changes in electrical properties of aging cells. Using cardiac myocytes isolated from 3-, 6-, and 18-month-old isogenic zebrafish, we examined the efficacy of MEDoS and showed that MEDoS can identify discernible changes in electrical impedance. Taken together, our data demonstrated that electrical impedance in cells at different ages is distinct with quantitative values; these results were comparable with previously reported ones. Therefore, we propose that MEDoS be used as a new biomarker-independent methodology to obtain quantitative data on the biological senescence status of individual cells.

## 1. Introduction

Senescence and disease are the two main contributing factors for the termination of life [1]. Although senescence is one of the major causative factors of disease, senescence can be controlled to extend lifespan. In this context, various biomarkers have been used to measure and analyze senescence. In particular, research on senescence is especially important in cardiovascular research because cardiac myocytes are long-lived postmitotic cells, which need renewal of cellular components as a major ability for lifespan, unlike other short-lived cell types [2].

In general, senescent cells have reduced autophagic activity [3], reduced telomerase activity [4], altered contents in mitochondrial phospholipid [5], increased oxidative stress due to reactive oxygen species (ROS) [6, 7], and increased levels of senescence associated *β*-galactosidase activity [8]. Additionally, senescence associated changes at various levels of gene transcription and protein translation have also been reported. In all of the aforementioned studies, specific biomarkers have been used to evaluate the potential alterations in cell structure and function. However, such analyses involve complex procedures including chemical modification or tagging. In addition, the acquired data provide only comparative (not absolute) values. Further, given that senescence is a highly complex biological process, it is difficult to assess cellular aging based on the limited number of available biomarkers.

Recently, changes in cellular components during senescence were quantitatively analyzed using a new methodology called microelectrochemical impedance spectroscopy for diagnosis of senescence (MEDoS), which involves measurement of electrical impedance of a cell. Since electrical properties of a cell gradually change with changes in the cellular components during senescence, cell impedance can be used to analyze senescence. In addition, cell impedance data can provide quantitative characteristic values for individuals with a higher efficiency than biomarkers. In this study, we investigated age-related changes in cell impedance in cardiac myocytes of zebrafish.

## 2. Materials and Methods

### 2.1. Animals

Three groups (3-, 6-, and 18-month-old) of zebrafish maintained at 30–40 fishes per 9-L tank with a 14/10 h light/dark cycle. The zebrafishes were fed living brine shrimps twice per day. Water was disinfected by using UV lamps to prevent the spread of diseases in the recirculating system. Water temperature was maintained at 28 ± 5°C. A continuously cycling aquatic habitat system maintained water quality (system type M, genomic design, Daejeon, Korea). Additionally, the system continuously circulated water from the tanks through a strainer into a chamber containing foam filters and activated carbon inserts. Water quality was tested daily for chlorine, ammonia, pH, and nitrate levels. The health of each fish was observed daily. All zebrafishes were fed in the same environmental system and all groups were genetically identical. This was ensured by screening the transgenic zebrafish at the embryonic stage.

### 2.2. Transgenic Zebrafish Heart Extraction,* Ex Vivo* Cell Dissociation, and Sorting

The necessity for sorting out cardiomyocytes in zebrafish (*Danio rerio*) led us to generate a Tg (cmlc2:EGFP) strain expressing EGFP (enhanced green fluorescent protein) under the control of the cmlc2 (cardiac myosin light chain 2) promoter, which specified GFP expression only in cardiac myocytes. Methods that we used for generating the transgenic fish were followed as described in the tol2kit transposon transgenesis [9]. Then the isogenic Tg (cmlc2:EGFP) was grouped as per their ages (3, 6, and 18 months old) in order to perform further experiments.

10–15 zebrafishes were selected to isolate cardiac myocytes from individual groups. After anesthetizing the animals by administering tricaine, the zebrafish hearts were dissected by using a knife and forceps. The dissected hearts were transferred to 15-mL tube containing Ca^2+^, Mg^2+^-free Dulbecco's phosphate buffer saline (D-PBS) (JBI, Gyeongsangbuk-do, Korea), and 2.5 mM ethylenediaminetetraacetic acid for 20 min at 4°C. This was followed by a quick wash with 5 mL of D-PBS thrice; the solution was partly removed to keep the volume under 200 *μ*L, and then 100 *μ*L of D-PBS with 60–100 *μ*L of Liberase DH Research Grade (Roche, Basel, Switzerland) was added. The solution was incubated at 29°C for approximately 15 min with occasional pipetting. Subsequently, 1 mL of 0.25% trypsin (JBI, Gyeongsangbuk-do, Korea) was added to the solution. After that, incubation was performed at 29°C for approximately 15 min with occasional pipetting. After trypsin treatment, 1% fetal bovine serum (JBI, Gyeongsangbuk-do, Korea) in 5 mL was added to inactivate the trypsin. Undissociated cell mass was removed by applying the solution to a 40 *μ*m cell strainer (BD Falcon, MA, USA). Dissociated cells were collected by centrifugation at 300 ×g at 4°C for 5 min and washed with 1 mL of 1% fetal bovine serum. Finally, cardiac myocytes were sorted by fluorescence-activated cell sorting (BD FACSAria III, Becton, D&C Company, Franklin lakes, New Jersey).

### 2.3. Microelectrochemical Impedance Spectroscopy for Diagnosis of Senescence (MEDoS)

Electrochemical impedance spectroscopy has been utilized to indicate the electrical characteristics of different types of tissues [10]. Even though the measurement of electrical impedance of tissues can provide beneficial information, this method is inconsistent and imprecise owing to the complex structure and composition of tissues [11]. Recently, microelectrochemical impedance spectroscopy has been developed to characterize the electrical properties of cells at the single-cell level owing to the advances in lab on a chip and microfabrication technologies [12, 13]. The electrical impedance measurement at the single-cell level can afford more precise information than that of measurements at the tissue level [11]. This technique contributed to acquire the quantitative information of cells, such as resistance, reactance, capacitance, and conductance, because the electric properties of cells are connected with their physiological states [14, 15].

Therefore, microelectrochemical impedance spectroscopy has been suggested to be a simple, fast, and cost effective diagnostic tool that does not require biomarkers. For example, quantification of cell impedance among different breast cancer grades [11], discrimination between normal and cancer cell of the prostate [16], and discrimination between normal and cancer urothelial cell [17] by using *μ*EIS have been reported. However, these previous studies just focused on discrimination and comparison within cell groups without explanation of mechanistic approach. To overcome this limitation, we developed microelectrochemical impedance spectroscopy for diagnosis of senescence (MEDoS).

### 2.4. Design of MEDoS

**Figure 1:**
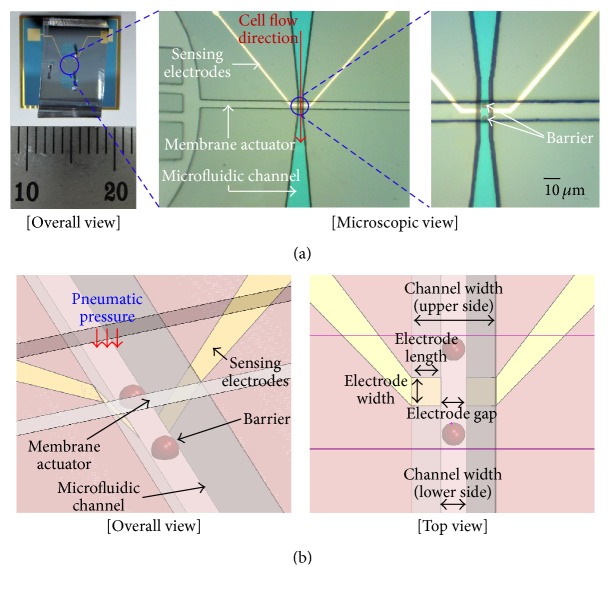
(a) Overall and microscopic view of microelectrochemical impedance spectroscopy for diagnosis of senescence (MEDoS) and (b) schematic overall and top view of the MEDoS.

MEDoS was designed to ensure that a captured single cell remains steadily at a certain position during measurement. The MEDoS comprises a microfluidic channel for cell flow, a flexible polymer membrane actuator that functions as a cell trap for capturing, a pair of barriers, and sensing electrodes (Figures 1(a) and 1(b)). Microelectromechanical systems technology was used to fabricate the MEDoS, whose specific dimensions are shown in Table 1.

The microfluidic channel has a tunnel structure. Both sidewalls of the microfluidic channel are formed with a slanted angle of 54.74° using a crystalline silicon wet etching process. As a result, the fabrication of the sensing electrodes is easy compared with that of the vertical sidewalls. The cell trap consists of the membrane actuator and a pair of barriers. The membrane actuator controls the cross-sectional area of the channel to effectively capture the cells by pneumatic pressure. Meanwhile, the height of the microfluidic channel is 15 *μ*m, which is slightly larger than the cell diameter, so that single cells can easily pass through the channel without cell clogging [16]. When 350 kPa of pneumatic pressure is the membrane actuator, the membrane actuator is inflated to reduce the cross-sectional area of the microfluidic channel. Nevertheless, certain cells easily leak out of the trap, which implies that structural barriers are additionally necessary to position a single cell at the center of the sensing electrodes with a high probability. In this study, a pair of barriers under the membrane actuator were fabricated at the front and back sides of the trap to enhance the capturing capability of cells. The barriers (height: 5 *μ*m) were made of negative photoresist, whose top part was rounded by thermal reflow to prevent the barrier edges from damaging the cells during trapping.

### 2.5. Experimental Setup

**Figure 2:**
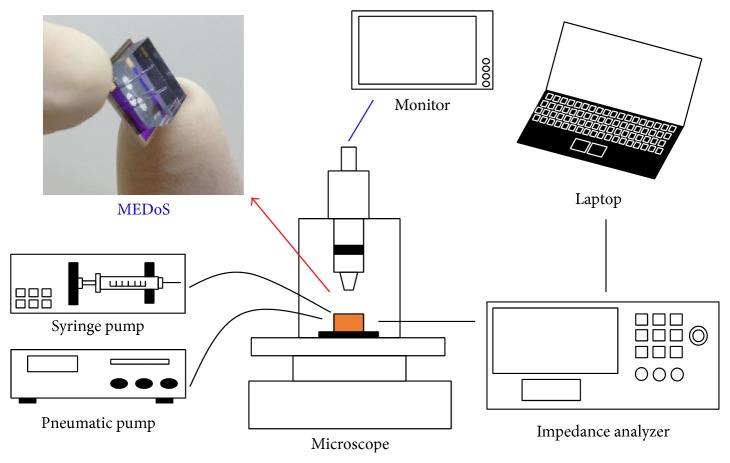
Experimental setup for measurement of electrical impedance for a single cell.

The electrical impedance was measured for the three groups of zebrafish cardiac myocytes by coupling MEDoS to a system that comprised an impedance analyzer (Reference-600, Gamry, PA, USA), a laptop, a pneumatic pump (EFD 1500-XL), and a syringe pump (Pump 11 Elite) (Figure 2). 350 kPa of pneumatic pressure was applied to the membrane actuator to position each cell onto the electrodes. Then, the impedance analyzer provided the MEDoS with a measuring voltage, the frequency of which was swept for 2 points per decade from 1 kHz to 1 MHz. The syringe pump regulated the flow rate at 0.1 *μ*L/min. The passage of cells in the microfluidic channel was monitored using a microscope (Eclipse L200, Nikon, Tokyo, Japan).

### 2.6. Measurement of Cell Impedance

The electrical impedance was measured for the captured cells (30 cells/group) at 350 kPa. The three groups of cardiac myocytes in 1% fetal bovine serum solution were infused into the microfluidic channel. The densities of the sorted 3-, 6-, and 18-month-old cardiac myocytes were under 5.62 × 10^4^ in 0.2 mL, 5.74 × 10^4^ in 0.2 mL, and 5.46 × 10^4^ in 0.2 mL of 1% fetal bovine serum solutions, respectively.

**Figure 3:**
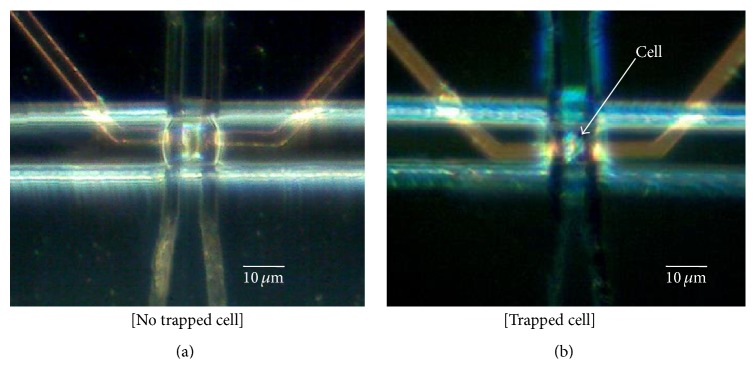
(a) Microscopic view of MEDoS when pneumatic pressure is applied to the membrane actuator. (b) The captured single cell is shown between the electrodes. Note: pictures are taken using reflective light source.

MEDoS performed in this study exhibited a high cell-capture rate (90%) for cardiac myocytes from zebrafish hearts. The sequence of cell trapping is as follows. (1) Three groups (3, 6, or 18 months old) of cardiac myocytes in 1% fetal bovine serum solution are injected into the fluidic channel. (2) The membrane actuator is inflated by pneumatic pressure to block the cell flow until a single cell stops in front of the trap. (3) The pressure is reduced so that a single cell can enter the trap in a squeezed state. (4) When a single cell is positioned at the center of the sensing electrodes, the pneumatic pressure is increased again to fix the cell on the central surface of the electrodes (Figures 3(a) and 3(b)).

A tight electrical contact between the cell and the sensing electrodes can be achieved by operating the membrane actuator. In addition, an electric field can be confined in a single cell because the width of the sensing electrodes is smaller than the length of the cell that is elongated by the membrane actuator. These conditions enable achieving a high measurement sensitivity with MEDoS to obtain necessary information from cells. For minimization of cell damage, cardiac myocytes were maintained at 4°C during the experiment, and all experiments were completed within 1 h.

Measured cell impedance was presented in terms of magnitude and phase angle for all frequencies. To specify electrical cell properties, resistance and capacitance were extracted through an equivalent electrical circuit model for single cell [18].

### 2.7. Statistics Analysis

All statistical calculations were carried out using PASW Statistics 18 (SPSS Inc., USA), and statistical analyses were performed using one-way ANOVA with* post hoc* tests (Scheffe and Games-Howell), Kruskal-Wallis test, and Mann–Whitney *U* test with Bonferroni correction.

## 3. Results 

### 3.1. Impedance Changing Patterns of Cardiac Myocytes of Different Ages

**Figure 4:**
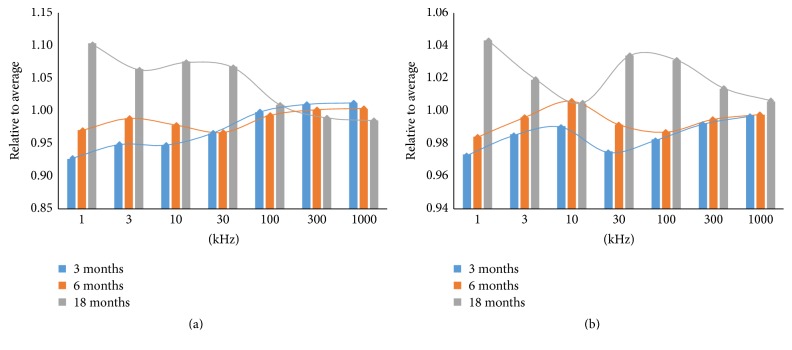
Electrical impedance with respect to frequency for zebrafish cardiac myocytes at different ages, presented as relation to the average value. (a) Magnitude and (b) phase angle.

In this study, changes in cell impedance during senescence in the three different age groups of zebrafish were evaluated in terms of magnitude and phase angle at the measured frequencies. The cells were the same type and differed only in age, and the three groups showed different patterns of changes in cell impedance, in both magnitude and phase angle. To compare the changing patterns of electrical impedance among the three groups, the ratio of the average value to the frequency for each group was calculated for magnitude (Figure 4(a)) and phase angle (Figure 4(b)). The measured values of cell impedance are listed in Table 2. There were statistically significant differences among the three groups at all frequencies, except the magnitude at 100 kHz (*p* < 0.001; one-way ANOVA).

### 3.2. Optimal Frequency for the Best Discrimination Capability

**Figure 5:**
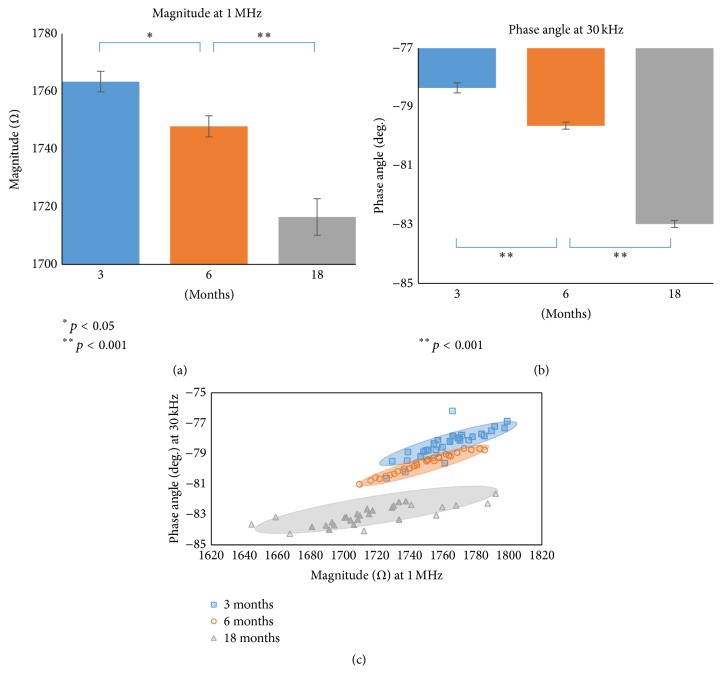
(a) Comparison of magnitudes at the optimal frequency (1 MHz) among the three age groups. Vertical bars represent standard error. (b) Comparison of phase angles at the optimal frequency (30 kHz) among the three age groups. Vertical bars represent standard error. (c) Distribution of the magnitude at 1 MHz versus the phase angle at 30 kHz for 30 cells from each group.

We determined the optimal frequency at which the difference in impedance for the three groups was highest in terms of *p* value. Moreover,* post hoc* tests confirmed a significant difference between groups (3- versus 6-month-old, 6- versus 18-month-old, and 3- versus 18-month-old groups) at the optimal frequency. Therefore, 1 MHz and 30 kHz were identified as optimal frequencies for the magnitude (Figure 5(a)) and the phase angle (Figure 5(b)), respectively. At 1 MHz, the average magnitude values for 3-, 6-, and 18-month-old cardiac myocytes were measured as 1762.78 ± 3.54 Ω, 1747.40 ± 3.62 Ω, and 1716.25 ± 6.28 Ω, respectively. Meanwhile, the average phase angles for 3-, 6-, and 18-month-old cardiac myocytes at 30 kHz were measured as −78.37 ± 0.17°, −79.67 ± 0.12°, and −83.06 ± 0.12°, respectively. The distribution of each group for the optimal frequency is shown in Figure 5(c).

### 3.3. Specific Cellular Values for Electrical Impedance, Resistance, and Capacitance

**Figure 6:**
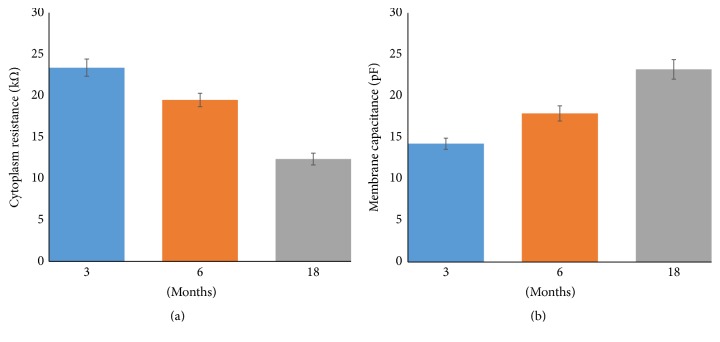
(a) Characteristic resistance of the cytoplasm and (b) capacitance of the cell membrane, depending on the age.

Through electrical circuit fitting model [18], the characteristic resistance of cytoplasm and capacitance of cell membrane were extracted from the measured cell impedance for each group of the cardiac myocytes. The estimated resistance of the cytoplasm decreased during senescence, being 23.25 ± 5.63 kΩ, 19.38 ± 4.43 kΩ, and 12.30 ± 3.85 kΩ in the 3-, 6-, and 18-month-old cardiac myocytes, respectively (*p* < 0.05; Kruskal-Wallis test and Mann–Whitney *U* test with Bonferroni correction; Figure 6(a)). On the other hand, the estimated capacitance of the membrane increased during senescence, being 14.81 ± 3.83 pF, 18.59 ± 5.20 pF, and 24.12 ± 6.69 pF in the 3-, 6-, and 18-month-old cardiac myocytes, respectively (*p* < 0.05; Kruskal-Wallis test and Mann–Whitney *U* test with Bonferroni correction; Figure 6(b)).

## 4. Discussion

Electrical impedance, one of the main electrical properties, is the ratio of applied voltage to induced current in the frequency domain, and it can be expressed by either magnitude and phase angle or resistance and reactance [19]. The magnitude is the absolute value of the voltage to current ratio, while the phase angle is the phase shift of the current compared to the voltage [19]. In our results, changes in cell impedance among the three groups were found in terms of magnitude and phase angle, with statistically significant differences. Specifically, the distribution of the three groups was clearly discriminated at the optimal frequencies. Such changes in electrical impedance can be explained by the fact that cellular components vary both in the cytoplasm and in the cell membrane during senescence [3–8], which eventually changes the electrical impedance of the cell [18, 20, 21]. Thus, the correlation between senescence and changes in electrical impedance should be investigated further.

To evaluate the correlation between cell impedance and changes in cellular components, an equivalent electrical circuit model for a single cell was used to extract specific electrical values of cellular components from the measured magnitude and phase angle. A simple model for an equivalent electrical circuit for a single cell can be based on the resistance (reciprocal value of conductance) of the cytoplasm and capacitance of the cell membrane [22]. Conductance will be used to indicate how easily electric current flows through a single cell, whereas the capacitance defines how rapidly electric charges are accumulated on a cell membrane [23]. Specifically, the cytoplasm was regarded as a conductive material that can be expressed by conductance because it consists of a highly conducting ionic solution with a high concentration of dissolved organic material. On the other hand, the cell membrane was considered a dielectric material that provides a capacitive path because it consists of a thin phospholipid bilayer with low conductance [22].

As shown in Figure 6(a), the resistance of the cytoplasm gradually decreased from the 3-month-old cell group to the 18-month-old cell group. Considering that resistance is inversely proportional to conductance, we reviewed previous aging studies that evaluated changes in cellular components that could affect conductance during senescence. Autophagic activity is especially important in cardiac myocytes, a long-lived postmitotic cell, to maintain homeostasis and longevity [24]. Autophagic activity decreases with senescence [25], and, accordingly, various ROS accumulate in the cytoplasm of cardiac myocytes [24]. Thus, accumulated ROS could cause changes in cellular components as well as in electrical impedance. In several studies, an increase in the conductance of induced hypoxic alveolar epithelial cells due to an increase in the ROS level was found [26]. In addition, an increase in the conductivity of hemoglobin caused by high oxidative stress was addressed [27]. In other words, accumulated ROS can increase the conductance of the cytoplasm because of their free-radical characteristics. Therefore, our results could suggest that ROS that accumulate during senescence decrease the resistance of the cytoplasm.

Meanwhile, capacitance (Figure 6(b)), which refers to the cell membrane in the electrical circuit model, gradually increased from the 3-month-old cell group to the 18-month-old cell group. A cell membrane has a phospholipid bilayer, which is composed of different types of molecules such as fatty acids and various proteins. During cell senescence, the level of ROS gradually increases with decreasing autophagic activity [3]. ROS are more soluble in the fluid lipid bilayer than in aqueous solution; thus, the membrane phospholipids and polyunsaturated fatty acids, one of the phospholipid acyl chains, are susceptible to oxidative damage [28]. Peroxidation of polyunsaturated fatty acids in the membrane has been shown to be a cause of senescence [29].

Based on the aforementioned studies, the peroxidizability index (PI) was used to measure the relative age-related susceptibility of fatty acid composition to peroxidative damage in the cell membrane. A high PI value implies that the membrane bilayer is easily affected by lipid peroxidation. Many investigators have found that the PI value and lipoxidation-derived molecular damage increase with aging [29]. In addition, the oxide composition amount increases during the process of lipid peroxidation [30]. These phenomena can be explained by the fact that high PI values are obtained as the oxide composition amount increases. The capacitance of the cell membrane also increases as the oxide composition amount increases in the membrane [18, 21]. Therefore, we hypothesize that the increase in PI values reflects an increase in the capacitance of the cell membrane.

Our approach to measuring cell impedance accurately requires a cell to be in close contact with the electrodes. In a previous study [16], a higher pressure facilitated close contact between cells and electrodes. We showed that single cells could be captured at 350 kPa but not at 150 kPa or lower. Thus, data on cell impedance that are more accurate can be obtained under higher pressure. Here, we used an advanced device (MEDoS) containing two barriers in the microfluidic channel, which improved the cell-capturing capability and accuracy of positioning of the cell between electrodes. With MEDoS, the interference between a target cell and subsequent cells was minimized compared to that in the device without barriers. In addition, the enhancement of the capturing capability when using a pair of barriers was proven in our recent study [18].

Meanwhile, our method of capturing cells with the membrane actuator has an advantage over other methods that do not include cell capturing in that the measurement accuracy is independent of cell size variation. The same type of cell could differ in size at different ages [31], which might reduce the measurement accuracy of electrical impedance. However, when a single cell was captured with the pneumatic pump, the captured single cell between the two barriers covers the entire width of the electrodes, minimizing variation in cell impedance regardless of cell size.

In this study, to minimize other variations that affect senescence, genetically identical, transgenic zebrafishes were used and were maintained in the same environment. However, further studies may be required to enhance the accuracy of measurement by this methodology. Future experiments should be carried out on the senescent change of mammal cells and also include* in vivo* tests to determine ways to minimize the effect of devascularized cells on the changes in their electrical properties [23], and sex-specific tests should be carried out to improve the discrimination capability of cell impedance [32].

## 5. Conclusion

We demonstrated that electrical impedance of a cell could be used as a quantitative index to analyze senescence in cardiac myocytes of genetically identical transgenic zebrafish. The* ex vivo* experimental results indicate that MEDoS could highly distinguish between the three age groups in this study. Especially, the optimal frequencies of magnitude and phase angle for the best discrimination capability were found to be 1 MHz and 30 kHz, respectively. Meanwhile, resistance of the cytoplasm monotonously decreased during senescence. On the contrary, capacitance of the cell membrane monotonously increased. This implies that conductance of the cytoplasm increased with accumulation of ROS, and progressive cell-membrane oxidation increased the capacitance of the membrane. In conclusion, cell impedance as a physical marker can provide quantitative cellular information, which could complement the existing biomarkers for research on senescence and disease progression.

## Figures and Tables

**Table 1:** Dimensions of the MEDoS.

Microfluidic channel for cell flow	Height (*µ*m)		15
	Width (*µ*m)	Upper side	30
		Lower side	10

Barriers in a trap	Height (*µ*m)		5
	Width (*µ*m)		8

Sensing electrodes	Gap (*µ*m)	Gap between electrodes on the bottom side of the channel	10
	Width (*µ*m)	Line width of an electrode	10
	Length (*µ*m)	Electrode length on the slanted sidewalls	18

**Table 2:** Measured cell electrical impedance (magnitude and phase angle).

Impedance	Frequency	3 months old (*n* = 30)	6 months old (*n* = 30)	18 months old (*n* = 30)	*p* value of 3 groups
Magnitude (Ohm)	1 kHz	10.00*E* + 05 ± 1.63*E* + 05	10.48*E* + 05 ± 1.19*E* + 05	11.91*E* + 05 ± 1.80*E* + 05	<0.001
	3 kHz	34.08*E* + 04 ± 2.74*E* + 04	35.54*E* + 04 ± 3.05*E* + 04	38.25*E* + 04 ± 3.20*E* + 04	<0.001
	10 kHz	11.59*E* + 04 ± 1.03*E* + 04	11.97*E* + 04 ± 0.51*E* + 04	13.16*E* + 04 ± 1.24*E* + 04	<0.001
	30 kHz	43.74*E* + 03 ± 1.45*E* + 03	43.83*E* + 03 ± 1.52*E* + 03	48.37*E* + 03 ± 2.06*E* + 03	<0.001
	100 kHz	16.02*E* + 03 ± 0.37*E* + 03	15.93*E* + 03 ± 0.40*E* + 03	16.19*E* + 03 ± 0.50*E* + 03	0.058
	300 kHz	53.79*E* + 02 ± 0.89*E* + 02	53.34*E* + 02 ± 0.90*E* + 02	52.70*E* + 02 ± 1.23*E* + 02	<0.001
	1 MHz	17.63*E* + 02 ± 0.19*E* + 02	17.47*E* + 02 ± 0.20*E* + 02	17.16*E* + 02 ± 0.34*E* + 02	<0.001

Phase angle (degree)	1 kHz	−81.63 ± 1.32	−82.54 ± 0.92	−87.46 ± 1.48	<0.001
	3 kHz	−82.94 ± 1.31	−83.81 ± 0.87	−85.73 ± 1.26	<0.001
	10 kHz	−81.08 ± 1.30	−82.30 ± 0.83	−82.19 ± 1.21	<0.001
	30 kHz	−78.37 ± 0.96	−79.67 ± 0.68	−83.06 ± 0.66	<0.001
	100 kHz	−81.77 ± 0.71	−82.15 ± 0.62	−85.79 ± 0.65	<0.001
	300 kHz	−83.55 ± 0.53	−83.74 ± 0.52	−85.31 ± 0.61	<0.001
	1 MHz	−79.68 ± 0.43	−79.71 ± 0.40	−80.36 ± 0.54	<0.001

## References

[1] Lakatta E. G. (2015). So! What's aging? Is cardiovascular aging a disease?. *Journal of Molecular and Cellular Cardiology*.

[2] Terman A., Brunk U. T. (2005). Autophagy in cardiac myocyte homeostasis, aging, and pathology. *Cardiovascular Research*.

[3] Brunk U. T., Terman A. (2002). The mitochondrial-lysosomal axis theory of aging: accumulation of damaged mitochondria as a result of imperfect autophagocytosis. *European Journal of Biochemistry*.

[4] Yang J., Chang E., Cherry A. M. (1999). Human endothelial cell life extension by telomerase expression. *Journal of Biological Chemistry*.

[5] Almaida-Pagán P. F., Lucas-Sánchez A., Tocher D. R. (2014). Changes in mitochondrial membrane composition and oxidative status during rapid growth, maturation and aging in zebrafish, Danio rerio. *Biochimica et Biophysica Acta-Molecular and Cell Biology of Lipids*.

[6] Abete P., Napoli C., Santoro G. (1999). Age-related decrease in cardiac tolerance to oxidative stress. *Journal of Molecular and Cellular Cardiology*.

[7] Manke A., Wang L., Rojanasakul Y. (2013). Mechanisms of nanoparticle-induced oxidative stress and toxicity. *BioMed Research International*.

[8] Minamino T., Miyauchi H., Yoshida T., Tateno K., Kunieda T., Komuro I. (2004). Vascular cell senescence and vascular aging. *Journal of Molecular and Cellular Cardiology*.

[9] Kwan K. M., Fujimoto E., Grabher C. (2007). The Tol2kit: a multisite gateway-based construction kit for Tol2 transposon transgenesis constructs. *Developmental Dynamics*.

[10] Rigaud B., Morucci J.-P., Chauveau N. (1996). Bioelectrical impedance techniques in medicine—part I: bioimpedance measurement second section: impedance spectrometry. *Critical Reviews in Biomedical Engineering*.

[11] Han A., Yang L., Frazier A. B. (2007). Quantification of the heterogeneity in breast cancer cell lines using whole-cell impedance spectroscopy. *Clinical Cancer Research*.

[12] Gawad S., Cheung K., Seger U., Bertsch A., Renaud P. (2004). Dielectric spectroscopy in a micromachined flow cytometer: theoretical and practical considerations. *Lab on a Chip*.

[13] Yong H., Ning C., Borninski J., Rubinsky B. A novel microfluidic cell-chip for single cell analysis and manipulation.

[14] Faes T. J. C., van der Meij H. A., De Munck J. C., Heethaar R. M. (1999). The electric resistivity of human tissues (100 HZ–10 MHZ): a meta-analysis of review studies. *Physiological Measurement*.

[15] González-Correa C. A., Brown B. H., Smallwood R. H. (1999). Virtual biopsies in Barrett's esophagus using an impedance probe. *Annals of the New York Academy of Sciences*.

[16] Kang G., Kim Y.-J., Moon H.-S. (2013). Discrimination between the human prostate normal cell and cancer cell by using a novel electrical impedance spectroscopy controlling the cross-sectional area of a microfluidic channel. *Biomicrofluidics*.

[17] Park Y., Kim H. W., Yun J. (2016). Microelectrical impedance spectroscopy for the differentiation between normal and cancerous human urothelial cell lines: real-time electrical impedance measurement at an optimal frequency. *BioMed Research International*.

[18] Park Y., Cha J.-J., Seo S. (2016). Ex vivo characterization of age-associated impedance changes of single vascular endothelial cells using micro electrical impedance spectroscopy with a cell trap. *Biomicrofluidics*.

[19] Irwin J. D., Nelms R. M. (2008). *Basic Engineering Circuit Analysis*.

[20] Matthews G. G. (2002). Electrical properties of cells. *Cellular Physiology of Nerve and Muscle*.

[21] Stark G. (2005). Functional consequences of oxidative membrane damage. *The Journal of Membrane Biology*.

[22] Das D., Kamil F. A., Biswas K., Das S. (2014). Evaluation of single cell electrical parameters from bioimpedance of a cell suspension. *RSC Advances*.

[23] Halter R. J., Schned A., Heaney J., Hartov A., Paulsen K. D. (2009). Electrical properties of prostatic tissues: I. Single frequency admittivity properties. *Journal of Urology*.

[24] Linton P.-J., Gurney M., Sengstock D., Mentzer R. M., Gottlieb R. A. (2015). This old heart: cardiac aging and autophagy. *Journal of Molecular and Cellular Cardiology*.

[25] Leon L. J., Gustafsson Å. B. (2016). Staying young at heart: autophagy and adaptation to cardiac aging. *Journal of Molecular and Cellular Cardiology*.

[26] Caraballo J. C., Yshii C., Butti M. L. (2011). Hypoxia increases transepithelial electrical conductance and reduces occludin at the plasma membrane in alveolar epithelial cells via PKC-*ζ* and PP2A pathway. *American Journal of Physiology—Lung Cellular and Molecular Physiology*.

[27] Moussa S. A., Abdelhalim M. A. K., Alhadlaq H. A. (2009). Evaluation of electrical conductivity of hemoglobin and oxidative stress in high fat diet rabbits. *Journal of Applied Sciences*.

[28] Yu B. P., Suescun E. A., Yang S. Y. (1992). Effect of age-related lipid peroxidation on membrane fluidity and phospholipase A_2_: modulation by dietary restriction. *Mechanisms of Ageing and Development*.

[29] Naudí A., Jové M., Ayala V., Portero-Otín M., Barja G., Pamplona R. (2013). Membrane lipid unsaturation as physiological adaptation to animal longevity. *Frontiers in Physiology*.

[30] Girotti A. W. (1985). Mechanisms of lipid peroxidation. *Journal of Free Radicals in Biology and Medicine*.

[31] Rubin H. (1997). Cell aging in vivo and in vitro. *Mechanisms of Ageing and Development*.

[32] Arslan-Ergul A., Adams M. M. (2014). Gene expression changes in aging Zebrafish (Danio rerio) brains are sexually dimorphic. *BMC Neuroscience*.

